# Utilization of Uterine and Umbilical Artery Doppler in the Second and Third Trimesters to Predict Adverse Pregnancy Outcomes: A Nigerian Experience

**DOI:** 10.1089/whr.2021.0058

**Published:** 2022-02-28

**Authors:** Ademola J. Adekanmi, Adebola Roberts, Imran O. Morhason-Bello, Abiodun O. Adeyinka

**Affiliations:** ^1^ Department of Radiology, College of Medicine, University of Ibadan, Ibadan, Nigeria.; ^2^ Institute of Cardiovascular Diseases, College of Medicine, University of Ibadan, Ibadan, Nigeria.; ^3^ Department of Obstetrics and Gynaecology, and College of Medicine, University of Ibadan, Ibadan, Nigeria.; ^4^ Institute of Medical Research and Advanced Training, College of Medicine, University of Ibadan, Ibadan, Nigeria.

**Keywords:** adverse pregnancy outcomes, second/third trimester, umbilical, uterine artery, Doppler ultrasonography

## Abstract

***Objective:*** To assess the utility of uterine and umbilical artery Doppler in the second and third-trimester in predicting adverse pregnancy outcomes.

***Methodology:*** In a prospective longitudinal study, the demographic, clinical, Doppler ultrasound parameters of the uterine and umbilical arteries of 84 consecutive women attending the antenatal clinic at 22–24 weeks and 116 women at 30–34 weeks gestation and pregnancy outcomes were documented and analyzed.

***Results:*** Pregnant women with adverse pregnancy outcomes had significantly higher second-trimester mean uterine systolic/diastolic (S/D) ratio (*p* = 0.001), pulsatility index (PI; *p* = 0.003), umbilical artery S/D (*p* = 0.016), and resistivity index (RI; *p* = 0.041) as well as higher third-trimester uterine S/D and PI. While pregnancies with adverse fetal outcomes showed significantly higher uterine artery S/D and PI at the second trimester, third-trimester uterine showed higher S/D, RI, and PI and umbilical artery PI than in women with normal fetal outcomes. The combination of uterine PI and early diastolic notch were predictors of maternal outcomes and correctly predicted 73% (*p* < 0.001) in the second trimester. By the third trimester, the uterine PI alone was the best predictor and accurately predicted about 62% of maternal outcomes (*p* = 0.028). In addition, the second-trimester uterine S/D and early diastolic notch and uterine PI in the third trimester correctly predicted 79% and 78% of fetal outcomes, respectively.

***Conclusion:*** Among unselected pregnant women population, the second-trimester Doppler parameters are better predictors of maternal adverse pregnancy outcomes, while adverse fetal outcome prediction by uterine and umbilical Doppler at the second- and the third-trimester parameters are comparable.

## Introduction

Complications of pregnancy are often associated with an adverse event in the mother and the fetus.^1^ Such complication may arise from preexisting maternal conditions that predate the pregnancy (chronic medical disorders and hemoglobinopathy) and previous obstetric problems. Others are maternal social and anthropometric characteristics (age, obesity) and health problems (pregnancy-induced hypertension, preeclampsia, eclampsia, anemia, and gestational diabetes) diagnosed during pregnancy, labor, and delivery.^1–3^

Other health problems resulting from complications of impaired placentation, such as preeclampsia, placental abruption, intrauterine growth restriction, and oligohydramnios, may lead to maternal and fetal complication deaths.^4^ It is also possible that uncontrolled gestational diabetes in pregnancy can cause unexplained fetal death, childbirth complications, increased risk of operative vaginal delivery, and early neonatal death.^5^

Generally, the hemodynamic changes in pregnancy are usually characterized by declining uterine and umbilical artery flow resistance and increased blood flow with advancing gestational age. However, pathological circulatory changes typified by high-resistant circulation in pregnancy have been associated with an increased risk of adverse pregnancy complications.^6^ The risk of adverse pregnancy complications can be reduced in developing regions if quality diagnostic medical facilities are available for a timely prenatal screening. This will help obstetricians offer well-informed interventions at the appropriate time to reduce perinatal morbidity and mortality.

Doppler ultrasound evaluation of the uterine and umbilical arteries has become an important clinical tool in screening pregnant women who could develop adverse pregnancy complications or outcomes,^7–11^ as high uterine and umbilical Doppler impedance and decreased blood flow are proven findings in pregnancies with adverse complications.^12^

There are divergent opinions on the best time during pregnancy to perform a Doppler scan to predict adverse pregnancy outcomes.^6–8,13–17^ While some authors observed that the second and third trimester obstetric Doppler combination could predict adverse obstetric complications,^6,8^ other researchers reported high efficacy of Obstetric Doppler ultrasound to predict adverse pregnancy complications in all trimesters of pregnancy.^7,13^ However, most studies use the second trimester Doppler scan to predict adverse outcomes in pregnancy.^14–16^ Furthermore, there is no consensus on the best Doppler parameters to predict adverse obstetric complications.^17^

In low- and middle-income countries, a crucial issue is the high proportions of pregnant women that register late for antenatal care.^18–22^ This implies that a significant proportion of these women will not have early Doppler scans to screen for potential adverse pregnancy complications. Therefore, while it is desirable to encourage early booking for optimal antenatal care, it is equally important to evaluate the efficacy of third trimester Doppler ultrasound considering most Nigerian pregnant women's current peculiar attitude to book late for antenatal care.

Furthermore, in recent times, there has been reported late-onset fetal growth restriction and preecalmpsia that the first trimester uterine Doppler may not identify, hence the need for assessment in the third trimester.^23^ In addition, scientific literature on Doppler studies shows that the uterine artery alone has been the major focus of attention in many studies evaluating the second or third trimester of pregnancy in predicting adverse pregnancy complications.^6–8,12,13,15,17^

In this study, we evaluated the utilization of both uterine and umbilical artery Doppler in detecting adverse pregnancy outcomes in each of the second and third trimesters, as well as determine the best Doppler parameter(s) that best predict adverse maternal and fetal complications and determine their degree of predictability among an unselected population of pregnant women. The outcome of this study, we hope, might help to predict possible complications that could be averted by obstetrician's prompt intervention, influence clinical guidelines in Nigeria and other environments with similar obstetric history.

## Materials and Methods

This study was a continuation of a longitudinal study on Uterine and Doppler Ultrasound assessment among pregnant women referred for management at the University College Hospital, Ibadan, Nigeria. Institutional Review Board (IRB) approval was obtained from Oyo State, Research Ethical Review Committee, Department of Planning, Research and Statistics, Ministry of Health Oyo State (Approval number AD 13/479/701). We described other details in the previous publications.^24,25^ All subjects gave informed consent after the study was explained, including interpretation into local dialect in appropriate cases. We anonymized the subjects' details by using initials, and data were kept in a safe place.

For this study, the sample size was determined using the sample size calculation for cross-sectional studies^26^:

$$n={Z_{\alpha }}^{2}p\left(1-P\right)∕d^{2},$$

*Z_α_* = 1.96, *p* = proportion of women with adverse pregnancy outcomes (0.135)^27^ and *d* = precision (0.05). A 10% nonresponse rate of *n* gives a minimum sample size of 200 pregnant women. Consecutive eligible pregnant women were recruited into the study until the sample size was achieved.

In brief, pregnant women with only singleton gestation were recruited during their first antenatal visit and followed until delivery. Pregnant women with confirmed malformed fetuses, multiple pregnancies, those who could not remember their last menstrual period or without an early obstetric scan, and participants who did not deliver in our facility were excluded from the study.

Two certified Radiologists performed the scan independently at each visit, and both were blinded to the clinical findings and laboratory results of participants. The degree of agreement by both radiologists was evaluated and compared using the reports of the initial 10 patients who were scanned. The interobserver agreement was high (*k* = 0.92) in the pilot study.

The pregnancy outcomes were documented by the Obstetricians while blinded to the ultrasound scan results. The results of the Doppler scan at 22–24 weeks and the scan performed at 32–34 weeks were documented.

Ethical clearance for this study was sought and approved by the Oyo state ministry of health Ethics Review Committee.

### Clinical evaluation

The demographic and obstetric parameters, including the pregnancy outcomes at the termination of pregnancy or delivery, were documented by the obstetrician in a structured format tool.

In this study, the following pregnancy complications/adverse outcomes are (1) hypertension in pregnancy (a systolic blood pressure ≥140 mmHg and/or diastolic blood pressure ≥90 mmHg), (2) preeclampsia/eclampsia (hypertension after 20 weeks' gestation accompanied by at least one of the following: proteinuria; features of maternal organ dysfunction, with or without right upper quadrant or epigastric abdominal pain, neurological complications, and hematological complications; and uteroplacental dysfunction such as fetal growth restriction, abnormal umbilical artery Doppler waveform analysis, or stillbirth), (3) abruptio placenta, or (4) gestational diabetes (fasting blood sugar ≥92 mg/DL or 5.1 mmol/L or 2-hour postprandial plasma glucose ≥153 mDDdL or 8.5 mmol/L).

The following fetal complications/adverse outcomes include (1) abortion (expulsion of product of conception before the age of viability; 28 completed weeks in our environment), (2) stillbirth (delivery of a fetus after 28 weeks of gestation without sign of life), (3) preterm birth (delivery before 37 weeks of gestation), (4) low birth weight (newborn weighing ≤2.5 kg), and (5) neonatal intensive care admission.

### Ultrasonographic evaluation

Ultrasonographic examination was performed using a GENERAL ELECTRIC LOGIQ P5 ultrasound scanner (Korea) with a curved array 3.5–5.0 MHz transabdominal transducer.

All participants had a baseline obstetric scan to exclude fetal anomalies and multiple pregnancies at recruitment. In addition, Doppler ultrasonography of the uterine and umbilical arteries was performed at 22–24 and 32–34 weeks of gestation using a semirecumbent position with a slight lateral tilt to avoid inferior vena cava compression by the gravid uterus.

The uterine artery was identified using the color Doppler mode, as it crosses the external iliac artery, following Bramham et al.'s technique.^28^ Pulsed wave Doppler was then applied following the Doppler scanning parameters; wall filter of 50–60 Hz, angle of insonation below 20°, and a gate size of 2 mm over the uterine artery at about 1 cm below the crossover point to generate the spectral wave pattern.

In the umbilical artery Doppler evaluation, a free loop of the umbilical cord was identified when there was no fetal movement or uterine contraction. With the color and pulsed wave, Doppler interrogation of the umbilical artery generated the spectral waveform following the technique of Bramham et al.^28^ and the International Society of Ultrasound in Obstetrics and Gynaecology.^29^

The generated uterine and umbilical artery spectral waveform was traced using automatic tracing, while manual tracing was also utilized in appropriate cases. Only the impedance parameters (systolic/diastolic ratio, pulsatility index, and resistivity index [S/D ratio, PI, and RI]) that are not dependent on vessel tortuosity of adulation were considered in this study. The mean of three consecutive recorded spectral waveforms was documented for S/D, RI, and PI. The pregnancy outcomes were also documented at delivery or termination of pregnancy.

### Data analysis

The data were analyzed using the IBM SPSS (Statistical Package for the social sciences) statistics for windows, version 23.0 (IBM Corp., Armonk, NY). Frequency distributions were generated with appropriate graphs and tables. The student t-test was used to test the association between Doppler parameters and adverse pregnancy complications. The Doppler parameter that best predicts pregnancy outcomes was assessed using conditional logistic regression analysis.

Furthermore, the receiver operator curve was used to determine the predictability of adverse pregnancy complications in the second and third trimesters of pregnancy. Comparison of areas under the receiver operating characteristic (ROC) curves was made using VassarStats: website for Statistical Computation software downloaded from http://vassarstats.net/. The level of statistical significance was set at *p*-values, <0.05 in this study.

## Results

A total of 200 pregnant women—92 high-risk and 108 normal/low-risk pregnancies, participated in this study. The mean age of participants was 31.5 ± 4.36 years, while the modal age group was 30 to 34 years (40.5%). The majority of the pregnant women (36.5%) were nulliparous. Doppler scans were performed at 22–24 and 32–34 weeks gestational age for second and third-trimester pregnancies, respectively. A total of 84 (42.0%) and 116 (52.0%) participants had second and third-trimester Doppler scans, respectively. Maternal complications were seen in 43.0% of the total population, while 40.5% had fetal adverse pregnancy outcomes (Table 1).

**Table 1.** Demographic Characteristic of the Study Population

Variables	Frequency	%
Age		
<25	8	4.0
25–29	60	30.0
30–34	81	40.5
35 and above	51	25.5
Gravidity		
Primigravida	40	20.0
Gravida 2–3	90	45.0
Gravida 4 and above	70	35.0
Parity		
Nulliparous	73	36.5
Primiparous	71	35.5
Multiparous	56	28.0
Nature of pregnancy		
High-risk pregnancy	92	46.0
Normal-/low-risk pregnancy	108	54.0
Trimester at scan		
Second trimester	84	42.0
Third trimester	116	58.0
Maternal pregnancy outcome		
Normal	115	57.0
Adverse	86	43.0
Fetal pregnancy outcome		
Normal	119	59.5
Adverse	81	40.5

### Doppler parameters at second and third trimester and adverse pregnancy outcomes

There was a significant difference in the second trimester mean uterine S/D ratio (2.03 ± 0.44 and 2.49 ± 0.97, *p* = 0.001), the mean uterine PI (0.78 ± 0.27 and 1.29 ± 0.79, *p* = 0.001) and the mean umbilical RI (0.59 ± 0.14 and 0.69 ± 0.14, *p* = 0.003) between women without and those with adverse maternal outcomes, respectively. However, the uterine artery RI, the umbilical artery S/D, and PI were not significantly different in the two groups. The majority of the pregnant women with early uterine artery diastolic notch 16 (57.1%) had adverse maternal outcomes, *p* = 0.011.

In the third trimester, there was a significant difference in the mean uterine artery S/D (1.86 ± 0.33 and 2.36 ± 1.02, *p* = 0.001), uterine PI (0.72 ± 0.21 and 1.08 ± 0.54, *p* < 0.001), and umbilical artery PI (0.86 ± 0.27 and 0.96 ± 0.25, *p* = 0.038) among women without and those with adverse maternal outcomes, respectively. However, the mean uterine artery RI, the umbilical artery mean S/D, and mean RI in the third trimester showed no significant difference between the two groups (Table 2).

**Table 2.** Association Between Pregnancy Outcome and Obstetric Doppler Velocimetry

Variables	Adverse maternal outcome		*p*
	No	Yes	
	Mean ± SD	Mean ± SD	
Second trimester Doppler			
Mean UT S/D	2.03 ± 0.44	2.49 ± 0.97	0.001
Mean UT RI	0.54 ± 0.14	0.56 ± 0.15	0.568
Mean UT PI	0.78 ± 0.27	1.29 ± 0.79	0.001
Mean umbilical S/D	2.55 ± 0.56	3.09 ± 1.42	0.075
Mean umbilical PI	1.07 ± 0.47	1.21 ± 0.57	0.228
Mean umbilical RI	0.59 ± 0.14	0.69 ± 0.14	0.003
Early UT diastolic notch			
No	40 (71.4)	16 (28.6)	
Yes	12 (42.9)	16 (57.1)	0.011
Third-trimester Doppler			
Mean UT S/D	1.86 ± 0.33	2.36 ± 1.02	0.001
Mean UT RI	0.48 ± 0.15	0.53 ± 0.16	0.070
Mean UT PI	0.72 ± 0.21	1.08 ± 0.54	<0.001
Mean umbilical S/D	2.30 ± 0.54	2.50 ± 0.69	0.080
Mean umbilical PI	0.86 ± 0.27	0.96 ± 0.25	0.038
Mean umbilical RI	0.54 ± 0.11	0.57 ± 0.09	0.114

PI, pulsatility index; RI, resistivity index; S/D, systolic/diastolic; SD, standard deviation; UT, uterine.

### Uterine and umbilical Doppler parameters and fetal outcomes

In the second trimester, there was a significant difference in the mean uterine S/D ratio (2.03 ± 0.49 and 2.76 ± 0.93, *p* < 0.001) and the mean uterine PI (0.80 ± 0.29 and 1.31 ± 0.82, *p* = 0.003) between normal pregnancies and pregnancies with adverse fetal complications, respectively. However, there was no difference in the umbilical artery impedance parameters between the two groups.

In third trimester, there was significant difference in the mean uterine S/D ratio (1.80 ± 0.35 and 2.46 ± 0.98, *p* < 0.001), RI (0.46 ± 0.15 and 0.55 ± 0.15, *p* = 0.002), and the mean uterine artery PI (0.70 ± 0.21 and 1.13 ± 0.52, *p* < 0.001) and mean umbilical artery PI (0.85 ± 0.24 and 0.98 ± 0.28, *p* = 0.009) between normal pregnancies and pregnancies with adverse fetal complications, respectively. However, there were no significant differences in other Doppler parameters between the two groups (Table 3).

**Table 3.** Association Between Perinatal Outcome and Obstetric Doppler Velocimetry

Variables	Adverse fetal outcomes		*p*
	No	Yes	
	Mean ± SD	Mean ± SD	
Second trimester Doppler			
Mean UT S/D	2.03 ± 0.49	2.76 ± 0.93	<0.001
Mean UT RI	0.53 ± 0.15	0.59 ± 0.12	0.067
Mean UT PI	0.80 ± 0.29	1.31 ± 0.82	0.003
Mean umbilical S/D	2.57 ± 0.67	3.12 ± 1.39	0.085
Mean umbilical PI	1.09 ± 0.46	1.18 ± 0.61	0.511
Mean umbilical RI	0.61 ± 0.14	0.66 ± 0.15	0.109
Early UT diastolic notch			
No	41 (73.2)	15 (26.8)	
Yes	14 (50.0)	14 (50.0)	0.035
Third trimester Doppler			
Mean UT S/D	1.80 ± 0.35	2.46 ± 0.98	<0.001
Mean UT RI	0.46 ± 0.15	0.55 ± 0.15	0.002
Mean UT PI	0.70 ± 0.21	1.13 ± 0.52	<0.001
Mean umbilical S/D	2.28 ± 0.52	2.53 ± 0.71	0.052
Mean umbilical PI	0.85 ± 0.24	0.98 ± 0.28	0.009
Mean umbilical RI	0.55 ± 0.09	0.57 ± 0.12	0.398

### Doppler parameters and predictors of adverse maternal outcomes

In the second trimester, the odds of adverse maternal outcomes were 13.6 times (95% confidence interval [CI] odds ratio [OR] = 2.57, 72.5; *p* = 0.002) and 3.60 times (95% CI OR = 1.14, 11.4; *p* = 0.030) more likely for a unit increase in the mean uterine PI and the presence of an early diastolic notch, respectively.

The odds of reporting an adverse maternal outcome in the third trimester were 12.4 times more likely (95% CI OR = 3.22, 47.8; *p* < 0.001) (Table 4).

**Table 4.** Association Between Pregnancy Outcome and Obstetric Doppler Velocimetry

Doppler parameters	AOR	95% CI	*p*
Second trimester			
Mean UT PI	13.6	2.57–72.5	0.002
Diastolic notch			
No	1		
Yes	3.60	1.14–11.4	0.030
Third trimester			
Mean UT PI	12.4	3.22–47.8	<0.001

AOR, adjusted odds ratio; CI, confidence interval.

**FIG. 1.**
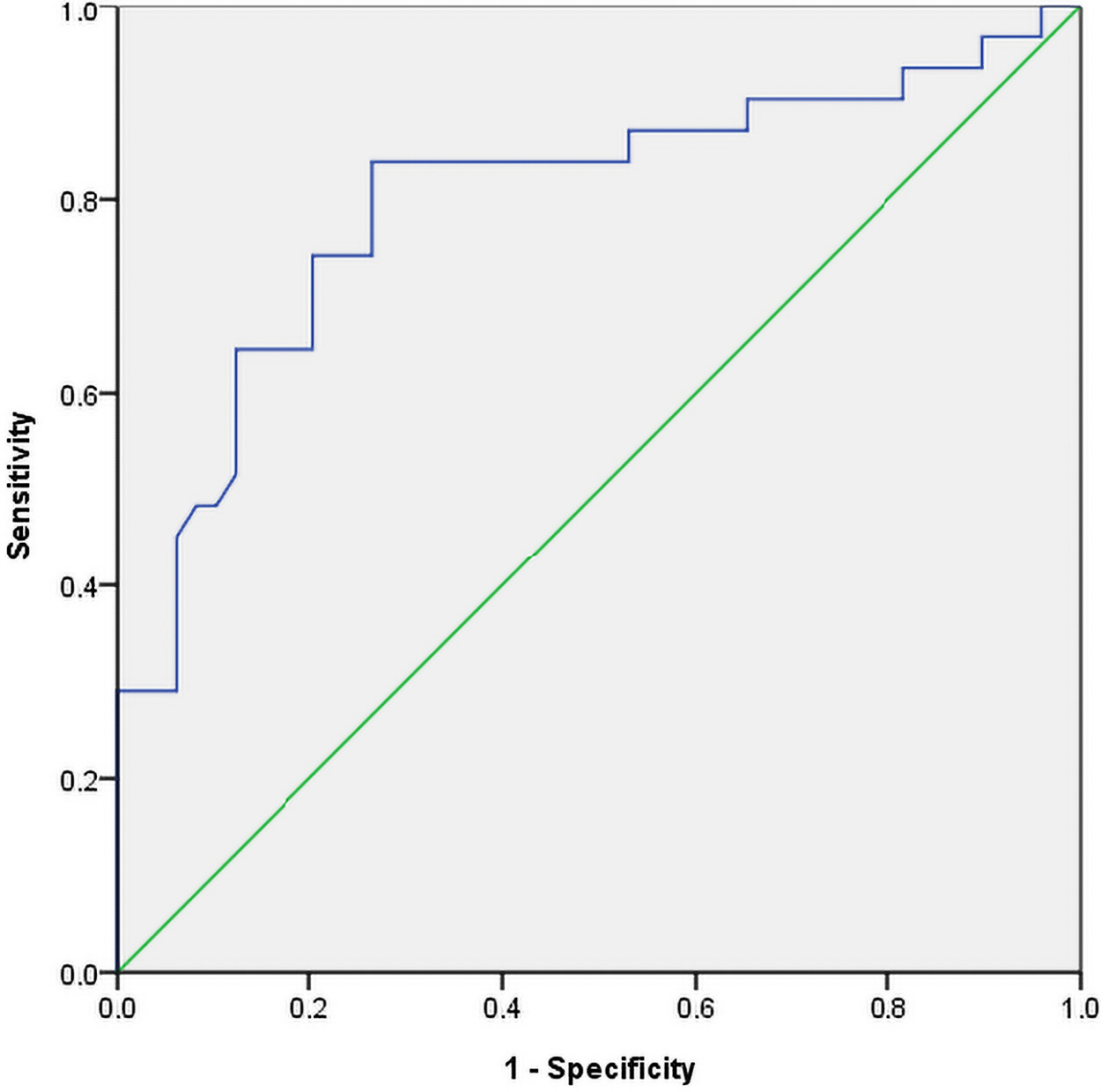
Receiver operating characteristic curve for the prediction of maternal complications using a combination of the second-trimester mean UT PI, and early diastolic notch. PI, pulsatility index; UT, uterine.

**FIG. 2.**
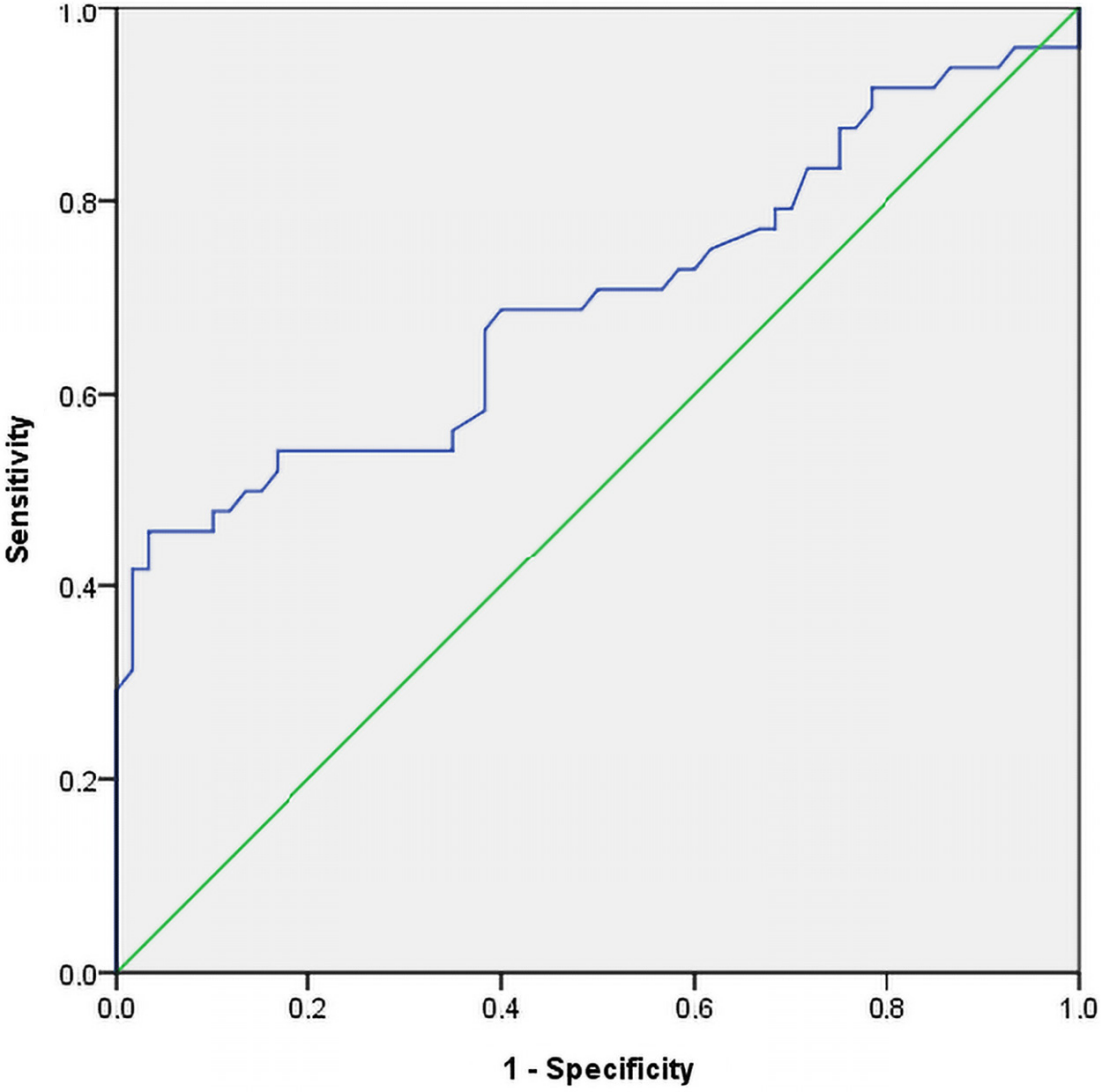
Receiver operating characteristics curve for predicting maternal complications using the third trimester mean uterine PI.

The receiver operating characteristic curve showed that the mean uterine PI and the early diastolic notch, in the second trimester, jointly predicted about 79.9% of maternal outcome correctly (Area Under Curve [AUC] = 0.799, 95% CI 0.690, 0.907; *p* < 0.001) (Fig. 1). In the third trimester, the mean uterine artery PI, predicted 69.6% of maternal outcome correctly (AUC = 0.696, 95% CI AUC 0.591, 0.801; *p* < 0.001) (Fig. 2).

Using a cutoff of the 95th percentile for PI (PI ≥1.40 and PI ≥1.0 in the second and third trimester, respectively),^30^ the sensitivity and specificity of using a combination of PI and a presence of early diastolic notch to predict adverse maternal outcomes in this study were 64.5% and 75.5% in the second trimester, while in the third trimester, the sensitivity and specificity of using PI ≥1.0 to predict adverse maternal outcomes in this study were 47.9% and 90.0%, respectively. However, using the Youden Index cutoff of PI ≥1.0 and PI ≥0.93 were obtained in the second and third trimester, respectively, to predict adverse maternal outcomes.

Thus, in the second trimester, the sensitivity and specificity of using a combination of PI ≥1.0 and the presence of early diastolic notch to predict adverse maternal outcomes in this study were 67.7% and 85.7%, respectively. In contrast, in the third trimester, the sensitivity and specificity of using PI ≥0.93 to predict adverse maternal outcomes in this study were 55.6% and 83.9%, respectively.

### Doppler parameters and prediction of adverse fetal outcomes

In the second trimester, the odds of reporting adverse fetal outcomes were 3.41 times (95% CI OR = 1.50, 7.77; *p* = 0.003) and 3.14 times (95% CI OR = 1.00, 9.81; *p* = 0.049) more likely for a unit increase in the mean uterine S/D ratio and a presence of an early diastolic notch, respectively. However, in the third trimester, the odds of adverse fetal outcomes were 4.75 times (95% CI OR = 1.48, 15.9; *p* = 0.012) for a unit increase in the uterine artery PI (Table 5).

**Table 5.** Multivariate Analysis of the Association Between Perinatal Outcome and Obstetric Doppler Velocimetry

Doppler parameters	AOR	95% CI	*p*
Second-trimester Doppler			
Mean UT S/D	3.41	1.50–7.77	0.003
Diastolic notch			
No	1		
Yes	3.14	1.00–9.81	0.049
Third-trimester Doppler			
Mean UT PI	4.75	1.48–15.9	0.012

**FIG. 3.**
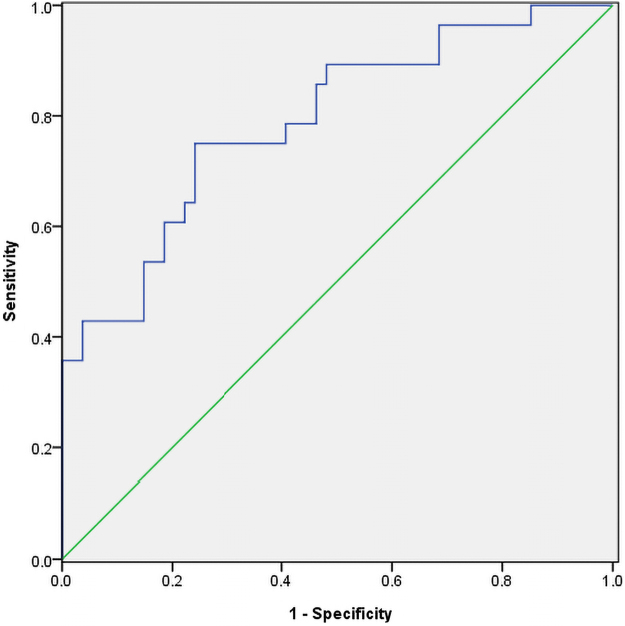
Receiver operating characteristic curve to predict perinatal complications using a combination of the second trimester mean UT artery early diastolic notch, and UT artery S/D ratio. S/D, systolic/diastolic.

**FIG. 4.**
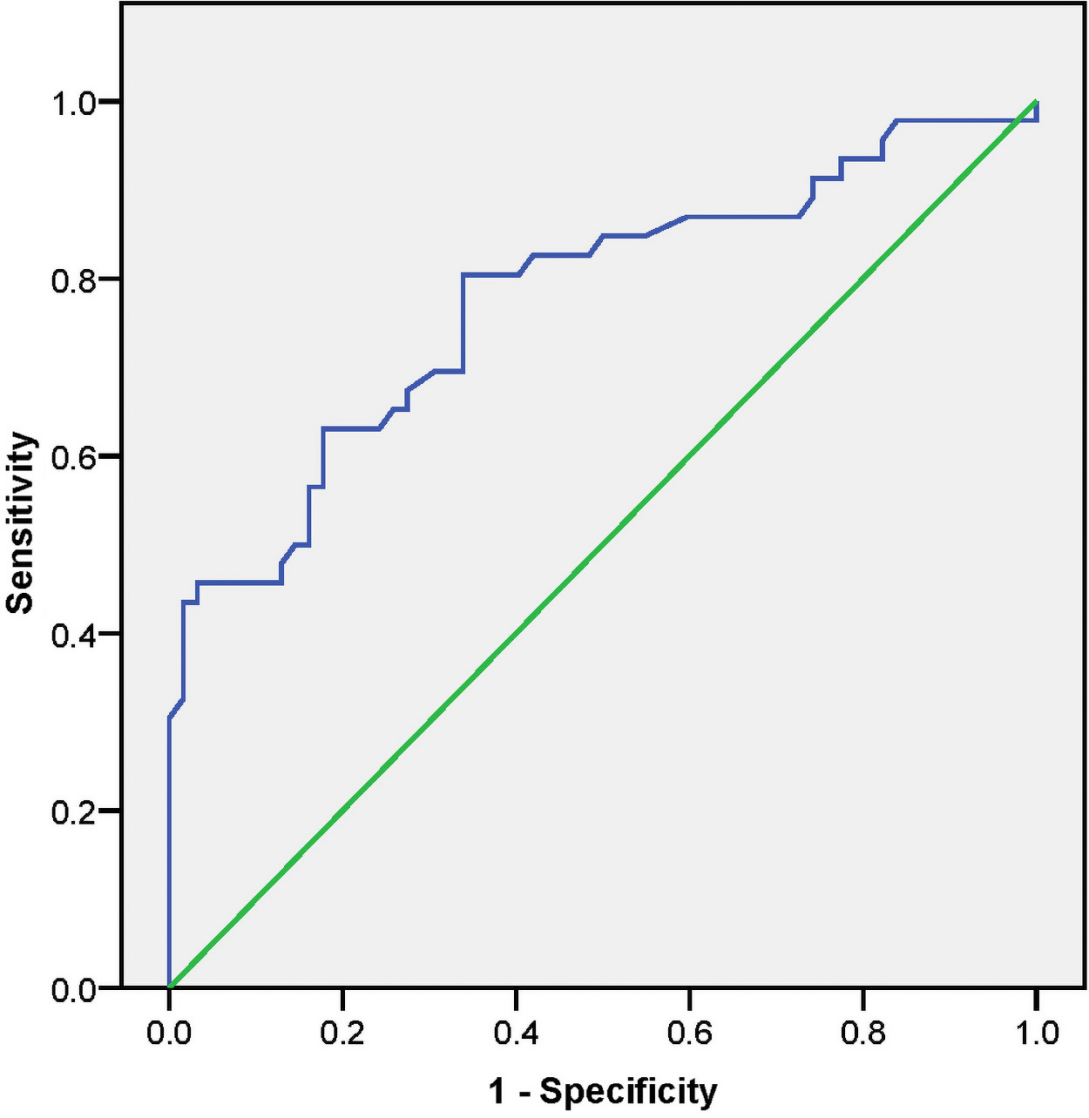
Receiver operating characteristic curve to predict perinatal complications using the third trimester mean UT artery PI.

The uterine artery S/D ratio and early diastolic notch significantly predict about 79% of fetal outcomes correctly (AUC = 0.790, 95% CI 0.686, 0.895; *p* < 0.001) in the second trimester (Fig. 3). Moreover, the third-trimester uterine artery PI correctly predicts 78% of fetal outcomes (AUC = 0.776, 95% CI 0.685, 0.868; *p* < 0.001), as shown in Figure 4.

Furthermore, using a cutoff of S/D ratio ≥2.60 in the second trimester,^31^ and PI ≥1.0 in the third trimester.^30^ The sensitivity and specificity of using a combination of S/D ratio ≥2.6 and a presence of early diastolic notch in the second trimester to predict adverse fetal outcomes in this study were 71.4% and 77.8%, respectively. In contrast, in the third trimester, the sensitivity and specificity of using a PI ≥1.0 to predict adverse fetal outcomes in this study were 45.7% and 87.1%, respectively.

However, using the Youden Index to select an appropriate cutoff for the Doppler parameters, an S/D ratio ≥2.58 and PI ≥0.87 were obtained in the second and third trimester, respectively, to predict the adverse fetal outcome. In the second trimester, the sensitivity and specificity of using a combination of S/D ratio ≥2.6 and an early diastolic notch to predict adverse fetal outcomes in this study were 71.4% and 77.8%, respectively, while the sensitivity and specificity of using a PI ≥0.87 to predict adverse fetal outcome in the third trimester in this study were 62.2% and 82.3%, respectively.

However, a comparison of the AUC curves showed no significant difference in the AUC of the second and third trimesters regarding adverse maternal (0.799 vs. 0.696; *p* = 0.153) and fetal outcomes (0.790 vs. 0.776; *p* = 0.845), respectively.

However, on multivariate analysis, other Doppler parameters did not significantly correlate with adverse maternal or adverse fetal outcomes.

## Discussion

This study evaluated the use of uterine and umbilical artery Doppler parameters at the second and third trimester of pregnancy to predict the adverse maternal and fetal complications among Nigerian women with a singleton gestation.

Similar to previous studies,^6,9,32,33^ elevation of the uterine and umbilical artery impedance indices (S/D, PI, and RI) in the second or third trimester is associated with maternal and perinatal complications. Besides, bilateral early uterine diastolic notch waveform was also significantly associated with adverse pregnancy complications. These findings were in tandem with previous studies that reported that a decrease in uteroplacental and fetoplacental blood flow, an increase in impedance indices, or the presence of bilateral uterine arterial notches is associated with a significantly increased risk of adverse outcomes in pregnancy.^34–36^

Previous works had reported that the second trimester uterine and umbilical Doppler velocimetry, particularly in the early second trimester, better predicts adverse pregnancy outcomes than the third trimester Doppler velocimetry scan.^14–16^ Although the second trimester uterine and umbilical artery Doppler velocimetry in this present study had a slightly higher prediction rate, especially for maternal complications than in the third trimester, there was no statistically significant difference.

In contrast, Afrakhteh and coworkers,^8^ in a prospective study of 205 singleton pregnant women, had Doppler ultrasound in the second and third trimesters. The study showed that the mean uterine RI was the best predictor of adverse pregnancy outcomes with a higher sensitivity in the second-trimester scan than the third-trimester scan (77.2% vs. 58.0%). However, findings from the study also suggested that uterine artery Doppler may be a valuable screening tool for predicting various adverse outcomes in second and third trimesters.

A number of studies have reported the use of a single Doppler parameter to predict pregnancy complications.^6,12,32,36^ In this study, we further evaluated the predictability of the second and third trimester Doppler scan to assess maternal and fetal complications. Our study showed that in the second trimester, the combination of the mean uterine artery PI and the early diastolic notch and a combination of mean uterine S/D ratio and early diastolic notch best predict maternal complications and adverse perinatal complications, respectively.

The observations are in tandem with previous studies that reported that combining Doppler parameters to predict adverse pregnancy complications gives better results than a single Doppler parameter.^16,37,38^ Furthermore, these studies also confirmed that combining Doppler parameters to predict adverse pregnancy complications gives better results than a single Doppler parameter.

Similarly, Cnossen et al.^39^ reported that an increased PI with an early diastolic notch in the second trimester best predicted overall maternal complications in low-risk and high-risk patients. Also, Kleinrouweler et al.^13^ reported that the combination of mean PI or RI and bilateral notching is the best predictor for adverse pregnancy complications. All in agreement with the observation of this study that the combination of Doppler parameters is more appropriate in predicting adverse pregnancy complications.

On using a Doppler scan to predict fetal complications in the second trimester, a study reported that the S/D ratio >2.6 and a notch in the waveform might indicate that the pregnancy is complicated by stillbirth, premature birth, intrauterine growth retardation, and maternal preeclampsia.^31^

In another study that assessed adverse maternal and fetal outcomes among 56 pregnant women with systemic lupus erythematosus,^40^ the results showed that a combination of S/D ratio of greater than 2.6 and the presence of diastolic notch is an important prognostic factor for adverse obstetric outcomes such as fetal loss, fetal growth restriction, and preeclampsia.

In the third trimester, the mean uterine PI, out of all Doppler parameters studied, best predicted maternal complications, while the uterine PI among all the parameters investigated adequately predicted adverse perinatal outcomes. Several investigators have shown third trimester PI as a single parameter or in combination with other Doppler parameters to predict adverse pregnancy outcomes.^6,8,9,32^

Another study examined the uterine and umbilical artery parameters among the women with a high mean PI above the 95th percentile at 19–22 weeks and was followed up at 28 weeks (early third trimester).^41^ The study concluded that the persistent elevation of the Uterine artery PI in the third trimester and not elevated PI of the umbilical artery is associated with an increased risk of adverse pregnancy outcomes. The findings from this study are similar to what was reported in previous studies that high resistance in the uterine artery in the third trimester of pregnancy increases the risk of adverse pregnancy outcomes.^41–43^

Our findings from the derived Doppler parameter cutoff from this study shows that sensitivity and specificity of using the cutoff of PI >1.0 and S/D ratio >2.58 in the second trimester and PI >0.93 and PI >0.87 in the third trimester to predict adverse maternal, and adverse fetal outcomes, respectively, were comparable to the results obtained when the assigned 95th percentile cutoff for PI (PI >1.40 and PI >1.0 in second and third trimester, respectively) and second trimester S/D ratio >2.60. These findings showed that the uterine PI cutoff value obtained using the Youden index method in the second and third trimesters was less than the 95th percentile cutoff for PI from the study by Gómez et al.^30^

Previously, Casmod et al. had reported that in the second trimester, only one out of seven patients with preeclampsia had a PI value above the 95th percentile, while in the third trimester, two out of the seven patients had a PI value above the 95th percentile.^44^ Furthermore, our finding also suggests using different Doppler parameters and cutoffs for different adverse pregnancy outcomes. This observation is similar to what Rabiu and Abubakar^45^ reported by using second-trimester umbilical RI to predict adverse pregnancy outcomes and different optimum cutoff points for various hypertensive disorders following ROC.

The interpretations of findings from this study are limited. First, the study was not performed to make a head-to-head comparison of the second and third-trimester Doppler scan to assess adverse pregnancy outcomes. Second, the study participants were not randomly selected from the pool of antenatal patients seeking care during the study period.

## Conclusion

This study shows that both the second and third trimester Doppler scans can predict adverse maternal outcomes with better outcomes when the Doppler scan was performed in the second trimester. Furthermore, the third trimester uterine and umbilical Doppler ultrasound parameters showed a comparable prediction of adverse fetal outcomes of the second-trimester Doppler scan parameters. Therefore, we believe that using an antenatal Doppler scan may be beneficial in assisting obstetricians in diagnosing and predicting possible adverse outcomes promptly.

The observations of this study will be communicated first to the obstetricians at our institution to sensitize them about the possibility of using the Doppler parameters to predict adverse pregnancy outcomes. Second, leveraging on our result, we would work with other researchers to have a multicenter study in Nigeria, thereby sensitizing Obstetricians and Physicians in Nigeria and other climes like ours to the use of reported Doppler Indices to predict pregnancy outcomes, thereby instituting prompt management to avoid or reduce to the minimum adverse outcomes in pregnancy.

## Abbreviations Used

AUC: area under curve
CI: confidence interval
GA: gestational age
OR: odds ratio
PI: pulsatility index
RI: resistivity index
ROC: receiver operating characteristics
S/D: systolic/diastolic
SD: standard deviation
UT: uterine
